# A Radiologic Study of Cardiac Symphysis and Partial Adhesions of the Pericardium

**Published:** 1913-09

**Authors:** 


A Radiologic Study of Cardiac Symphysis and Partial Adhesions of the Pericardium. By H. Vaquez and E. Bordet. (Arch. des Mal. du Coeur, des Vaisseaux, et du Sang, 1913, VI, 1). The authors present an interesting study of the application of X-ray examinations to the diagnosis of pericardial adhesions, partial or complete. They have found the flouroscopic study of diaphragmatic movements of value in determining adhesions between the heart, the pericardium and the diaphragm, provided there is no pathologic condition affecting the lungs, the pleurae, or the liver. Adhesions at the base of the heart are indicated by irregular, serrated shadows along the superior border of the heart; immobility of the base of the heart when the patient turns to one or the other side; diminution of or absence of the displacements of the upper third of the cardiac shadow during respiration; and slight changes of the mobility of the diaphragm. Adhesions at the apex are indicated by shadows of the adhesive bands; and by immobility of the apex upon changing the position of the patient. Adhesions in the neighborhood of the diaphragm are indicated by marked diminution or complete absence of the movements of the entire diaphragm or of one side of that muscle. Adhesions to the entire thoracic wall are indicated by diminution or abolition of the movements of the heart dependent upon respiration or change of the position of the patient. In the lateral view the retrosternal clear space is dark during forced inspiration. Posterior mediastinitis is indicated by an abolition of the clear space behind the heart.

In cases in which the adhesions are situated in more than one location a combination of the above mentioned signs will be discovered. In comparing the signs furnished the diagnostician by radiographic examination and by percussion, the authors find that the right border of the heart can be located more accurately by the former than by the latter. The location of the apex beat and its degree of mobility, however, are quite as accurate by the one as by the other. Radiography is of particular value in the demonstration of the shadows of adhesions on the borders of the heart, respiratory displacements of the heart, changes in the movements of the diaphragm; obscuration of the anterior and posterior mediastina. Percussion, on the other hand, is of greater importance in determining the relation of the absolute to the relative cardiac dullness and in demonstrating the absence of influence on inspiration and expiration on the borders of cardiac dullness.

